# The Unequal Distribution of Linguistic Capital in a Transnational Economic Order

**DOI:** 10.3389/fsoc.2021.568962

**Published:** 2021-04-01

**Authors:** Jörg Rössel, Julia H. Schroedter

**Affiliations:** Department of Sociology, University of Zurich, Zurich, Switzerland

**Keywords:** linguistic capital, transnationalisation, Switzerland, economic approach to language, Bourdieu, labor market

## Abstract

Foreign language proficiency is an unequally distributed form of linguistic capital that is becoming increasingly important in contemporary societies: first, it enables persons to participate transnationally in educational activities and in labor markets beyond the national institutions of their home country. It is also crucial for integrating an increasing share of the population with a migration background into the labor market. Thus, this article focuses on the explanation of language proficiency. Its main aim is to enrich the discussion in this field by deriving hypotheses from the sociological theory of reproduction and the discourse on migrant integration. Variables are included which have not been tested in a broad fashion in previous empirical research. We use data on different groups of migrants and non-migrants in multilingual Switzerland, where we could study the determinants of the unequal distribution of language proficiency in three official languages and foreign language repertoire in general. Our main results show that the hypotheses derived from the two theoretical discussions are empirically supported overall and contribute substantially to the explanation of language proficiency. However, most of these variables indicate the importance of unequally distributed opportunities for learning languages, thus highlighting that language learning may be part of the general process of reproducing social inequality structures.

## Introduction

Foreign language proficiency is an unequally distributed and increasingly important resource in contemporary societies: on the one hand, it enables people to participate transnationally in educational activities and labor markets beyond the national institutions of their home country and profit from these boundary crossing engagements. On the other hand, it is crucial for the structural integration of an increasing share of the population with a migration background into both the labor market and the educational system of their country of residence. Up to now, the relevance of language proficiency has been discussed in two more or less completely separate strands of literature. The first is research focusing on the increasing importance of foreign language proficiency of transnationally mobile and active people in the era of globalization (e.g., Fligstein, 2008; Gerhards, 2010), while the second includes migration and integration research aimed at the language proficiency of people with a migration background (e.g., Van Tubergen and Kalmijn, 2005, 2009; Esser, 2006a). In both strands of literature, it is assumed that language proficiency is a form of cultural capital, in this case linguistic capital, which can be invested in education systems and especially in the labor market and occupations. This linguistic capital is also mostly acquired in educational institutions (Chiswick, 2008; Gerhards, 2010), giving an advantage to those with higher education. Hence, foreign language proficiency, or a broad repertoire of languages in general, is deeply intertwined with structures of social, economic, and occupational inequality. This is both true for migrants, who differ in their proficiency in the languages of their country of residence, and for transnationally active people, who usually have a higher endowment with linguistic capital compared to the rest of the population. In this article we try to connect these two strands of literature, assuming that the major determinants of language proficiency do not differ between migrants and transnationally active persons. With regard to language proficiency, we focus on the number of fluently spoken languages as the dependent variable, both for the national languages of Switzerland and foreign languages in general.

There is a broad range of literature on the conditions for learning a second or further language (which for convenience we simply call foreign languages) in different disciplines, focusing on psychological, linguistic, social, and biological foundations of language. Our paper concentrates on the social contexts of foreign language proficiency (measured as the number of languages in which a person is orally proficient) in order to determine foreign language proficiency's relationship to social and economic inequality and migration experiences. In our contribution we mainly draw on two theoretical discussions which have so far not had much impact in the discussion on language proficiency and its determinants: Pierre Bourdieu's sociological theory of reproduction, which reveals the structural inequalities underlying the unequal distribution of foreign language proficiency. This theory has rarely been tested in quantitative studies of language proficiency (Gerhards, 2010; Rössel and Schroedter, 2014). In addition, since proficiency in the languages of their destination countries is especially important for migrants' structural integration into the labor market and the occupational structure, we add theoretical concepts and hypotheses derived from migration and integration research, focusing especially on the ongoing discussion on the role of transnational ties and experiences for integration into the host country. We broaden this discussion by taking into account that transnational ties and experiences are not only a characteristic of migrant populations, but also of transnationally active autochthonous population segments (Dahinden, 2009; Mau, 2010). Our study goes beyond most previous research by accounting for a very differentiated set of measures of transnational experiences both for Swiss persons and migrants in Switzerland. As a kind of background theory we also briefly summarize the well-established economics of language approach, without deriving hypotheses from it, which conceptualizes foreign language proficiency as a kind of human capital and thus language learning as a form of capital investment. This approach is quite widespread not only in the literature on economics, but also in social science studies of migrants' proficiency in the languages of their host countries (Van Tubergen and Kalmijn, 2005, 2009; Esser, 2006a; Chiswick, 2008; Chiswick and Miller, 2014).

We aim to test the hypotheses derived from these two streams of theoretical discussions with respect to linguistic capital in the German-speaking part of Switzerland to enrich the existing discussion, which is strongly based on the economic approach on language learning. Furthermore, existing research on the social and economic determinants of foreign language proficiency usually focuses on only one destination country language. We take a step beyond this, since Switzerland is a country with four national languages (German, French, Italian, Rhaeto-Romanic). With regard to language integration, there is still the conviction in most cantons of Switzerland that pupils should learn another national language as their first foreign language and English as second foreign language. In some cantons, however, English is the first foreign language and another national language is the obligatory second foreign language (EDK, 2021).

An analysis of job advertisements in the German-speaking part of Switzerland shows that in 2015 and 2016, 14% of all positions required proficiency in French and 4% in Italian^1^. Furthermore, 24% of all job advertisements required knowledge of English. These language requirements are especially widespread in high-status occupations and in public administration. The actual use of different languages in the workplace is even higher: according to the Swiss Federal Statistical Office, in 2014, 28% of all people in paid work used more than one language at work on a daily basis [BfS (Bundesamt für Statistik), 2018, p. 7]. Economic transactions among the different language regions of Switzerland are more often conducted in one of the national languages than in English (Andres et al., 2005). Furthermore, the economic premium for being fluent in another national language is as high as the premium for being fluent in English (Grin, 1999). This means that both Swiss and foreigners increase their labor market opportunities by speaking more than one of the Swiss national languages fluently. In other words, speaking more than one national language is profitable for both persons with and without a migration background.

Accordingly, in our empirical study we do not focus only on proficiency in one language, but due to the specific situation in Switzerland, we study the determinants of linguistic capital in general (number of (foreign) languages spoken) as well as those of Swiss-specific linguistic capital (number of Swiss national languages spoken). In both cases, linguistic capital is an unequally distributed resource which is important not only for the higher rungs of the international labor market, but also for successful integration into the Swiss job market. Our empirical study thus contributes to the current state of research by taking a broad theoretical discussion and the hypotheses derived from it into account, focusing on a case in which proficiency in more than one language is relevant, and by studying persons with and without migration experiences. Hence, it goes beyond existing studies on linguistic capital in multilingual countries by first comparing migrants and autochthonous populations and then by studying both proficiency in national languages and (foreign) language competence in general (cf. Chiswick and Miller, 1994, 2001; Van Tubergen and Wierenga, 2011).

We proceed by presenting the main theoretical discussions we draw on and developing hypotheses based on the sociology of reproduction and the research on migration and integration. We then introduce our unique dataset based on a stratified random sample of migrants and non-migrants living in Zurich, Switzerland. These data enable us to study the language proficiency of persons with and without a migration background. Additionally, the data set is rich in relevant variables, enabling us to operationalise quite specific hypotheses derived from both theoretical discussions. Based on these data, we test the theoretical hypotheses developed before finally discussing our results and concluding with a short summary and outlook on further research.

## Theoretical Approaches

Our research contributes mainly hypotheses derived from two theoretical discussions: Bourdieu's theory of linguistic capital; and, the discussion concerning migration and the integration of migrants (cf. also Rössel and Schroedter, 2014). However, we start by briefly introducing the economic approach to language as a benchmark theory in the field. The two other perspectives may yet provide important additional insights and explanations for the unequal distribution of linguistic capital and its relationship to the economic and occupational structure of inequality.

### The Economics of Language

The economic approach to language considers language learning to be an investment in individual human capital (Chiswick, 2008; Chiswick and Miller, 2014). The main determinants are therefore economic incentives to learn a language, efficiency in learning a language, opportunities to practice and learn it and, finally, the costs of learning. A number of variables are related to *economic incentives*: for example, it can be assumed that migrants who want to stay in the host country for an extended period of time can make better use of their language skills in the national language than those who only plan to stay for a short period. It can also be assumed that migrants in higher professional positions can make better use of their language skills (Chiswick, 2008; Braun, 2010, p. 17–18; Chiswick and Miller, 2007; Isphording et al., 2014)^2^. Thus, higher earnings associated with certain occupations could be a major incentive to invest in destination language proficiency. The second key determinant of the economic model, the *efficiency of language learning*, describes the ability of individuals to translate formal and informal opportunities for language learning into de facto language competency. The most important variable at this point is the age at which learning begins, which has a strong influence on the ability to learn languages (Birdsong, 2006; Hufeisen and Riemer, 2010, p. 745). Furthermore, people with a higher level of education show higher efficiency in learning, as do people who already speak more than one language (e.g., people who grew up in bi-/multilingual families). A barrier to language learning efficiency is the linguistic distance between a person's first language and the second or further language (Chiswick, 2008, p. 14–17; Van Tubergen and Kalmijn, 2005; Hufeisen and Riemer, 2010, p. 745–747). The third determinant of *learning opportunities* is not only formal educational institutions that offer language learning opportunities, but above all opportunities to learn a language in everyday life, like vacations or longer stays abroad (Chiswick, 2008, p. 10–12; Braun, 2010; Chiswick and Miller, 2014).

The economics of language approach is empirically well-supported and thus forms a kind of standard perspective of language learning. Therefore, in our empirical study, we will control for several variables derived from this theory, like occupational status, employment status, linguistic distance between mother tongue and destination country language. However, the main focus of our article is on the hypothesis derived from the sociology of reproduction and the migration and integration literature, which will be discussed in the next section. These approaches hint at variables which have not been tested broadly in previous research.

### Theory of Reproduction

The economic approach to language conceptualizes language learning mainly as an individual and voluntary choice to invest in foreign languages. However, this view tends to overlook the structural bases and constraints of such investments and thus the relationship between inequalities in linguistic capital and the unequal distribution of other types of capital and thus the ongoing reproduction of inequality in society. Pierre Bourdieu, as the most important protagonist of the theory of reproduction, sees language proficiency as one form of cultural (linguistic) capital. Traditionally, especially proficiency in the legitimate standard language of a nation-state is a form of cultural capital which may be invested in the education system or the labor market (Bourdieu, 1990; cf. Gerhards, 2010; Rössel and Schroedter, 2014). This is an especially important form of linguistic capital for migrants, since the inequality of linguistic proficiency in the destination country language is ususally greater among migrants compared to persons without migration background. However, with contemporary conditions of Europeanisation and transnationalisation, this may have changed. Persons with proficiency in different foreign languages may have advantages in the educational system and the labor market (Fligstein, 2008; Gerhards, 2010). Thus, the value of language proficiency depends on the institutionalization of certain languages as legitimate in a certain society and beyond its borders. Therefore, in our study we focus not only on the standard languages of Switzerland, but also on the general foreign language repertoire (in doing so, we exclude the mother tongue). Bourdieu mentions three main sources of cultural capital^3^: practices, the education system and the parental home (Bourdieu, 1970). His assumption is that legitimate forms of cultural capital are mainly learned in the parental home and educational system. In this context, people usually acquire the legitimate culture of a society, such as the standard national languages and traditional “highbrow” culture, which is transferable to other fields and enables actors to grasp other and new forms of cultural capital. Thus, a highbrow cultural orientation should also further the acquisition of linguistic capital.

In Bourdieu's conception, societies' class structure results from the distribution of the types of capital. He sees the different classes and class fractions of capitalist society not only entangled in an economic class struggle, but also in a symbolic class struggle for the validity of values and culture. In this respect, the class structure of society is expressed in the various cultural lifestyles and language varieties. This means that the market in which investments take place is already oriented in favor of the dominant classes or the autochthonous population. Thus, the social position of the class factions with a surplus of cultural capital is reproduced by transferring this capital, in addition to the early acquisition of cultural competence, primarily through investments in the educational market and acquiring the highest possible academic titles (Bourdieu, 1982, p. 442–444). The habitus acquired in the early stages of socialization at home is fundamental to successful learning at school and acquiring academic titles. Pupils and students highly endowed with incorporated cultural capital are more versatile in their interests and have the ability to understand and enjoy works of classical high culture and to use the legitimate high-level language with stylistic confidence (Bourdieu and Passeron, 1977). It is precisely these skills that also come into play in the communication between professors and students and in the structure of exams, with the effect that the incorporated cultural capital is reflected in measurable educational successes (cf. Sullivan, 2001; Rössel and Beckert-Zieglschmid, 2002; Jaeger and Breen, 2016). Thus, the investment of linguistic capital is not only an individual, voluntary choice but part of a societal process of reproducing inequality. However, up to now only a few quantitative studies have tested Bourdieu's ideas on the reproduction of linguistic capital (Gerhards, 2010; Rössel and Schroedter, 2014).

Especially the role of the parents and of highbrow orientations have often not been studied in the economics of language approach. Whereas the relationship between highbrow orientation and language proficiency and repertoire is presumably not unidirectional, the impact of parents' education and language repertoire can be interpreted in a causal way, since it is not very likely that parents' education or language repertoire depends on the later language repertoire of their children. Based on the above considerations, we derive the following hypotheses from Bourdieu's theory of reproduction.

H_1_: The higher the parents' education, the larger the respondents' (foreign) language repertoire (transnational linguistic capital) will be, i.e., the number of foreign languages they speak fluently.
H_2_: The broader the parents' language repertoire, the larger the respondents' (foreign) language repertoire will be.
H_3_: The stronger the person's orientation toward classical highbrow culture, the larger their (foreign) language repertoire will be.
H_4_: The higher the person's education, the larger their (foreign) language repertoire will be.

An important similarity between the economic approach and Bourdieu's theory is the focus on opportunities to learn and practice a language. In contrast to the economic approach, Bourdieu emphasizes the inequality in opportunities leading to unequally distributed linguistic capital. This is relevant to the following discussion on the relationship between transnational experiences and linguistic capital, which highlights aspects that have received little attention in the literature so far. Derived from Bourdieu's emphasis on the importance of practice and the relevance of learning opportunities in the economic approach, our general assumption is that transnational experiences – enabling individuals to practice a given language – have a positive impact on linguistic capital.

### Migration, Transnational Experiences, and Integration

The relevance of transnational experiences and relations for the integration of migrants has been intensively discussed in the literature (e.g., Snel et al., 2006; Schans, 2009; Soehl and Waldinger, 2010). In more recent discussions, it has been acknowledged that not only migrants, but also autochthonous persons have transnational experiences and relations (Dahinden, 2009; Mau, 2010; Teney and Deutschmann, 2018). Thus, these experiences and practices may have an effect on both migrants, but also autochthonous Swiss' linguistic proficiency. According to the concept of exposure in the economic approach to language, such experiences and relations may also be relevant for language acquisition, since they are opportunities to learn and practice a new language. However, such transnational activities are often resource-based (Itzigsohn and Saucedo, 2002; Guarnizo et al., 2003; Portes et al., 2003; Fligstein, 2008; Gerhards, 2010). That is, they can also contribute to unequal opportunities in language learning. Furthermore, transnational experiences might have different effects on language acquisition for migrants and the autochthonous population – especially in respect to the Swiss-specific linguistic capital. Accordingly, we will first discuss the relationship between transnational experiences and integration in the host society for migrants, taking into account the special case of multilingual Switzerland (cf. Rössel and Schroedter, 2014). We will then broaden the discussion and derive a hypothesis applicable to the autochthonous population and the language repertoire in general.

The ability to speak the language of the host country is essential for acculturation and social integration. It also facilitates integration into other social subsystems, in particular the labor market (Esser, 2006a). Currently, there is not a great deal of systematic empirical research on the relationship between transnational relations/experiences and integration into host societies. The traditional theoretical perspective in assimilation research mostly regards transnational relations as incompatible with assimilation (Alba et al., 2002; Schans, 2009; Amelina, 2010). However, the results of empirical research on this question are somewhat inconclusive. Overall, the few general studies on transnational relations and integration do not show a clear pattern (e.g., Guarnizo et al., 2003; Portes et al., 2003; Snel et al., 2006; Soehl and Waldinger, 2010).

Concerning how transnational relations and activities relate to linguistic proficiency in the language of the destination country, the results of empirical studies are much more clear-cut. Having more social relations and experiences in the country of destination (spouse, previous stays, friends, interethnic networks) leads to higher language proficiency (Esser, 2006a,b, 2008; Van Tubergen and Kalmijn, 2009; Braun, 2010). The contrary is true for social relations and experiences with the country of origin. This also seems to hold for the language repertoire of non-migrants in foreign languages, i.e., the more international their social networks and experiences, the broader their language repertoire (Gerhards, 2010; Rössel and Schroedter, 2014).

In general, it must be assumed that the process of reproducing cultural and linguistic capital outlined by Bourdieu is less seamless among migrants than among persons without migration experience, as migration research emphatically shows (cf. Jacob and Kalter, 2011). Migrants are typically embedded in transnational social contexts, i.e., in their contexts of origin as well as in the contexts of the host country. As a result, in the context of the economic theory of language acquisition outlined above, certain factors may be disadvantageous for migrants when acquiring Swiss-specific linguistic capital. While, for example, educational qualifications, stays abroad and social contacts with foreigners can generally be advantageous for foreign language acquisition, in the case of migrants, these will often be directed toward the context of origin (educational qualifications outside Switzerland, transnational relations in the country of origin, social networks within their own ethnic group), so that knowledge of the mother tongue or other languages is generally deepened, but not necessarily knowledge of the Swiss national languages (Chiswick, 2008; Braun, 2010, p. 12; Stevens, 1985)^4^. We therefore expect that migrants will have a deeper linguistic competency in foreign languages in general, but not in the Swiss national languages – in particular if they are from countries where none of the Swiss main languages are spoken. Furthermore, given the importance of education and its effect on learning efficiency and exposure, we assume that education in the country of destination has a major positive impact on destination language skills.

H_5_: Traveling to foreign countries, social relations with persons in foreign countries and longer stays abroad have a positive impact on transnational linguistic capital.
H_6_: Persons with a migration background exhibit a smaller repertoire of national languages, i.e., Swiss-specific linguistic capital.
H_7_: Opportunities to speak the mother tongue have a negative impact on the repertoire of national languages.
H_8_: Education in Switzerland has a positive impact on Swiss-specific linguistic capital.

## Data and Methods

Our analysis is based on data from an online survey that we conducted in the context of the project “Toward a European Society: Single Market, Binational Marriages, and Social Group Formation in Europe (EUMARR)” between June and September 2012 in Zurich. The inquiry was addressed to persons in mono-national and binational partnerships (both marital and non-marital). The sample included persons from Switzerland, the EU-27 countries and other European and non-European countries that were living together with their partner. It is thus not a representative survey of the total population of Zurich, but a survey of a nationally and culturally very heterogeneous group that somewhat exaggerates the heterogeneity of Zurich's population. The basic sample was drawn randomly from several predefined strata of people from the population register of the city of Zurich^5^. We contacted all selected people by post in German and English and invited them to participate in the online survey. It was possible to answer the online survey both in German and English. With increasing time intervals, we sent three reminders to the sampled persons. The third and final reminder included a paper questionnaire which could be returned free of charge. This procedure yielded a response rate of about 40 percent.

Our dataset contains information on a rather select group of people in the population of Zurich. Due to the national and cultural heterogeneity of the sampled individuals, it is well-suited for analysing the causal mechanisms underlying the accumulation of linguistic capital. The advantage of the data set is the rich coverage of variables relating to social background and transnational experiences. This provides an opportunity to test hypotheses both for persons with and without a migration background. However, in interpreting the results one should take into account that the sample probably contains persons with an above-average endowment with transnational experiences and networks.

As a background to our sampling strategy one has to keep in mind that Switzerland's population contains roughly 25% foreigners, the majority of whom (over 80%) come from European countries. Switzerland adopted a treaty of free movement for EU citizens in June 2002, guaranteeing the freedom to move and work in Switzerland but also access for those commuting across the border to Switzerland (EDA, 2021). This results in Europeans having a fairly secure legal status in Switzerland. This legal situation combined with the strict naturalization laws may be the reason for the high percentage of foreigners in the Swiss population. With regard to labor market opportunities, migrants in Switzerland cover more or less all horizontal and vertical segments of the occupational structure. However, with increasing social and cultural distance from Switzerland, migrants face poorer labor market opportunities (Ebner and Helbling, 2016).

As mentioned above, we studied two different dependent variables. On the one hand, we considered the number of foreign languages spoken fluently (*transnational linguistic capital*), and on the other hand the number of official Swiss languages spoken fluently (*Swiss-specific linguistic capital*). Both variables were based on two questions in the survey. First of all, individuals were asked to indicate the language in which they were raised. It was possible to provide up to three answers, but respondents were invited to first list the language they would consider their mother tongue. Additionally, the respondents were asked to note the foreign languages they spoke fluently^6^. Due to overlaps in the questions and answers and pronounced multilingualism in Switzerland and especially in Zurich, it seemed advisable to merge the information of both questions. The maximum value of the variable of transnational linguistic capital was therefore set to four, allowing all respondents to give the same number of possible answers. Accordingly, the category applies to four and more fluently spoken (foreign) languages. The number of the national languages spoken was restricted to three, as Rhaeto-Romanic is only spoken by a small fraction of the population and all of those in our sample could also speak (Swiss) German. This means that in the first case (*transnational linguistic capital*), we added up all languages a respondent spoke minus the mother tongue; in the second case (*Swiss-specific capital*), we added up all national languages a respondent spoke, independent of their monther tongue (i.e., including the mother tongue for those with either (Swiss) German, French, or Italian as their mother tongue).

It has to be noted that the dependent variables have two weaknesses. First, they are merely a partial measure of language skills, as only the abilities to speak and to understand are captured, not the abilities to read and write. However, there is usually a strong correlation between the different elements of language competency (Jude, 2008). Additionally, in the Swiss labor market, oral use of the foreign language is the most important element, before reading and writing skills [BfS (Bundesamt für Statistik), 2018, p. 7]. Second, it is a subjective measure of language skills, i.e., the respondents had to indicate themselves whether they are able to speak a language or not. Although empirical research has shown that subjective and objective measures of language competencies correlate to a high degree (approx. *r* = 0.5), there is also a considerable chance of measurement error. Nevertheless, it has been shown that the measurement error does not influence the analysis of the determinants of language competencies extensively (Charette and Meng, 1994; Van Tubergen and Kalmijn, 2005).

In the next step, we will introduce the independent and control variables. First of all, we differentiate between (1) Swiss persons without and (2) Swiss persons with a *migration background* as well as (3) foreigners to test hypothesis 6. The differentiation between Swiss and foreigners is merely based on their formal citizenship(s). Swiss with a migration background are defined as Swiss citizens who meet one or more of the following criteria: (a) foreign citizenship(s) in addition to Swiss citizenship, (b) born abroad, (c) at least one parent born abroad. Sixty-three percent of such respondents were born in Switzerland. The vast majority of foreigners are first-generation migrants born abroad (92%) and come from one of the EU27 countries (88%). Sixty percent of the foreigners are citizens of one of the neighboring countries. Thirty-nine percent of all foreigners in the sample have German citizenship, 11% Italian, 7% Spanish, and 5% British. Of the Swiss with a migration background, 63% were born in Switzerland and 43% have an additional citizenship of an EU27 member state. Sixteen percent of all Swiss with a migration background have Italian as their second citizenship, 9% German. For the analysis of Swiss language capital, we further differentiate between foreigners who speak one of the main Swiss languages as their mother tongue and those who do not. We label Swiss citizens as autochthonous if neither of their parents were born abroad. Nevertheless, this group could include third generation migrants.

With regard to the *intergenerational reproduction of cultural capital*, we included the following variables: *education of father and mother*, the *number of foreign languages each parent spoke when the respondent was a child, highbrow cultural orientation*, and *education of the respondent*. As the majority of the respondents were highly educated, we distinguished between secondary education or less, postsecondary education and two levels of tertiary education. Higher tertiary education applied to persons who held a PhD or an equivalent degree. A *highbrow cultural orientation* was measured as an additive index whereby different regular highbrow cultural activities were summed up (going to classical music concerts, the theater, the opera, visiting museums, or exhibitions).

The migration context as well as *transnational relations and experiences* were operationalised as follows: for both analyses (transnational and Swiss-specific linguistic capital) we considered *regular contact with friends and relatives* (including in-laws) (a) within or (b) outside of the European Union. Regular contact meant that the respondent on average visited his/her friends and/or relatives in the respective region at least once a year. Both variables are dichotomous. Another variable concerns the *partner's mother tongue*. As the survey did not include this variable, it was based on the information of the partner's country of birth. The most frequently spoken language in the country was assumed to be the mother tongue. In the first analysis (all foreign languages), we only differentiated between partners speaking the same mother tongue as the respondent and those speaking a different one. In the second analysis of Swiss-specific linguistic capital, we further differentiated between partners with a different mother tongue who spoke a Swiss main language and those who spoke another language. Furthermore, we also took into account whether they were raised bilingually or multilingually. Moreover, we included a variable on *trips within Europe* as well as longer stays abroad. *Trips* referred to visits with at least one overnight stay and up to 3 months. *Longer stays abroad* related to visits that lasted at least 3 months. We only added up the number of visits to (or stays in) different countries. For the analysis of Swiss-specific linguistic capital, we modified both variables in that they only captured travels and stays abroad to countries where one of the main languages of Switzerland is spoken, namely German, French, or Italian. In this analysis, we considered one further variable: the *percentage of friends born in Switzerland within the respondent's (max.) five best friends in Switzerland*. Regarding hypotheses 7 we also included *being an English native speaker* and the *share of inhabitants in Zurich who speak the same mother tongue*. Both variables decreased the necessity to learn one of the Swiss national languages. And regarding hypothesis 8, we took into account whether persons *acquired the highest qualification in Switzerland*.

In respect to the *economic approach to language learning*, we take several empirically established constructs into account as *control variables*, especially being employed, occupation and the language family of the mother tongue. *Being employed* is a dichotomous variable discriminating between those currently in paid work and those not. *Occupation* is based on the major ISCO-08 codes and differentiates among managers, professionals, technicians and associate professionals, clerical support workers, service and sales workers, and a broader category of mainly trades and elementary occupations. People who were not employed at the time were asked to provide information on their last paid job. Individuals who had never worked before were rare in our sample (<3%) and were subsumed under the “missing” category. Our assumption based on the economics of language is that especially those in high-status occupations (managers and professionals) will benefit from foreign language skills and accordingly have a higher incentive to invest in learning another language. The *language family* is relevant insofar as individuals whose mother tongue belongs to the same language family as the Swiss main languages are expected to face lower costs in learning one of the respective languages. We differentiated between the main languages, Indo-Germanic languages and other languages. Age and sex were also included as control variables.

## Analyses

The language competencies of the respondents in our sample are comparatively high. Tables 1, 2 present the distributions of language competencies of the Swiss EUMARR survey for our two dependent variables, transnational and Swiss-specific linguistic capital, for Swiss and migrants.

**Table 1:** Language competencies in the sample: transnational linguistic capital.

	**Number of fluently spoken additional (foreign) languages**			
	**Swiss**	**Swiss with migration background**	**Foreigners**	**Total**
None	3%	1%	1%	2%
One	14%	10%	18%	14%
Two	36%	29%	38%	35%
Three	31%	33%	31%	31%
Four and more	16%	27%	13%	18%
Mean (SD)	2.4 (1.0)	2.8 (1.0)	2.4 (0.9)	2.5 (1.0)
Total	697	532	702	1,931

*Source: Data from the Swiss EUMARR survey*.

**Table 2:** Language competencies in the sample: Swiss-specific linguistic capital.

	**Number of fluently spoken Swiss** national languages^*^				
	**Swiss**	**Swiss with migration background**	**Foreigners with a main language as mother tongue**	**Foreigners with other mother tongue**	**Total**
None	–	1%	–	7%	1%
One	18%	18%	28%	59%	27%
Two	52%	43%	52%	27%	46%
Three	29%	38%	20%	7%	26%
Mean (SD)	2.1 (0.7)	2.2 (0.7)	1.9 (0.7)	1.3 (0.7)	2.0 (0.8)
Total	697	532	419	283	1,931

*Source: Data from the Swiss EUMARR survey*;

* *Rhaeto-Romanic included in German*.

On average, according to a broad survey of languages in Switzerland, Swiss speak two foreign languages (Werlen, 2008). Especially Swiss from the German-speaking and Italian-speaking language regions stand out, with 2.2 foreign languages, whereas French-speaking Swiss on average speak only 1.7 foreign languages (Werlen, 2008, p. 3). According to this comprehensive survey of the language situation in Switzerland, most Swiss persons also speak one of the other national languages (84% of the German Swiss, 62% of the French Swiss, and 88% of the Italian Swiss) (Werlen, 2008). The respondents of the Swiss EUMARR sample possessed slightly more transnational linguistic capital than the average Swiss, but the mean was close to that of the Swiss Germans. On average, the respondents spoke 2.5 foreign languages (cf. Table 1). Swiss with a migration background had the highest language competency: 27% of them spoke four or more languages. However, Swiss and foreigners in our sample did not differ much in their foreign language competency. Overall, the most frequently spoken foreign language was English (90%), followed by French (63%), Italian (27%), German (22%), and Spanish (21%) (cf. Table A1 in the Appendix for further details).

We find that on average, the respondents spoke two of the Swiss national languages (cf. Table 2). Swiss with a migration background also had the highest endowment with Swiss-specific linguistic capital. Foreigners with a mother tongue other than one of the main Swiss languages unsurprisingly had the lowest level of respective language proficiency. Almost all respondents spoke (Swiss) German (97%), while French was spoken by 67% and Italian by 34% (cf. Table A2).

In the following, we show the results of testing the hypotheses for linguistic capital accumulation using Poisson regressions with robust standard errors^7^. Table 3 shows the results for transnational linguistic capital, Table 4 the results for Swiss-specific linguistic capital^8^. In both cases, the models are structured as follows: the first model (1) contains the control variables age and sex, the migration background of the respondents and control variables relating to the economic approach of language learning (employment and occupations). We then add further variables step by step: first those relating to the intergenerational reproduction of cultural capital (model 2); and second, transnational experiences and relationships as well as further variables representing opportunities for learning and practicing languages (model 3). This allows us to systematically understand whether the variables derived from these two theoretical discussions increase the explanatory power of the statistical models.

**Table 3:** Determinants of the acquisition of transnational linguistic capital.

	**Model 1a**		**Model 2a**		**Model 3a**	
	**IRR**	**R.SE**	**IRR**	**R.SE**	**IRR**	**R.SE**
Age (cent. 37 years)	1.00	0.00	1.00	0.00	1.00	0.00
Sex (rf. male)	1.10^***^	0.02	1.11^***^	0.02	1.11^***^	0.02
Migration background (rf. Swiss)						
Swiss with migration background	1.13^***^	0.02	1.11^***^	0.02	1.04	0.02
Foreigner	0.97	0.02	1.00	0.02	0.98	0.03
Currently employed (rf. not employed)						
Employed	1.001	0.02	0.99	0.02	0.97	0.02
Occupation (rf. professionals)						
Missing	0.94	0.04	1.02	0.04	0.99	0.04
Managers	1.00	0.02	1.04	0.02	1.00	0.02
Technicians	0.90^**^	0.03	0.97	0.03	0.95	0.03
Clerical support workers	1.00	0.03	1.12^***^	0.03	1.09^**^	0.03
Service and sales workers	0.84^***^	0.03	0.94	0.03	0.94	0.03
Tradespeople	0.75^***^	0.05	0.84^**^	0.06	0.85^*^	0.05
Education of father (rf. sec. II)						
Missing			0.97	0.07	0.94	0.07
Secondary education I or less			1.07	0.04	1.03	0.03
Postsecondary			1.01	0.03	1.02	0.03
Tertiary			0.98	0.02	0.98	0.02
Education of mother (rf. sec. II)						
Missing			1.06	0.09	1.05	0.08
Secondary education I or less			1.05	0.03	1.03	0.03
Postsecondary			1.02	0.03	0.99	0.03
Tertiary			1.03	0.03	0.99	0.02
Father: no. of foreign languages			1.07^***^	0.01	1.07^***^	0.01
Mother: no. of foreign languages			1.02^*^	0.01	1.01	0.01
Education (rf. secondary ed.)						
Postsecondary			1.05	0.03	1.05	0.03
Tertiary I			1.16^***^	0.03	1.14^***^	0.03
Tertiary II			1.20^***^	0.04	1.15^***^	0.04
Highbrow cultural orientation			1.02^**^	0.01	1.01^*^	0.01
No. of trips to European countries					1.004^*^	0.00
No. of stays in different countries					1.04^***^	0.01
Partner with different mother tongue					1.14^***^	0.02
Social network within the EU					1.05^*^	0.02
Social network outside of the EU					1.05^*^	0.02
English native speaker (rf. no)					0.72^***^	0.04
Multilingual (rf. no)					1.12^***^	0.02
Percent of persons with same language					0.999^***^	0.00
Highest qualification in Switzerland					1.14^***^	0.03
Intercept	2.40^***^	0.07	1.71^***^	0.07	1.48^***^	0.08
Chi^2^	136.74		374.83		695.20	
Pseudo-R^2^ (Nagelkerke)	0.03		0.06		0.11	
AIC	6088.23		6050.28		5984.38	
BIC	6155.02		6194.99		6179.18	
N	1,931		1,931		1,931	

*Source: Data from the Swiss EUMARR survey*;

* *p < 0.05*;

** *p < 0.01*;

*** *p < 0.001*.

*How to read the table: The incident rate ratio (IRR) for a dichotomous variable is simply the ratio of the number of events of one category to the number of events in the other category. In model 1a it shows, for instance, that Swiss with a migration background are – ceteris paribus – expected to have a rate 1.13 times greater for the number of foreign languages than Swiss without a migration background. Each additional language a father speaks is associated with an estimated 7% increase in languages spoken by the respondent*.

**Table 4:** Determinants of the acquisition of Swiss-specific linguistic capital.

	**Model 1b**		**Model 2b**		**Model 3b**	
	**IRR**	**R.SE**	**IRR**	**R.SE**	**IRR**	**R.SE**
Age (cent. 37 years)	1.01^***^	0.00	1.01^***^	0.00	1.01^***^	0.00
Sex (rf. male)	1.09^***^	0.02	1.09^***^	0.02	1.08^***^	0.02
Migration background (rf. Swiss)						
Swiss with migration background	1.03	0.02	1.01	0.02	1.01	0.02
Foreigner with national language	0.91^***^	0.02	0.92^***^	0.02	0.92^**^	0.02
Foreigner	0.65^***^	0.02	0.65^***^	0.02	0.80^***^	0.04
Currently employed (rf. not employed)						
Employed	1.02	0.02	1.00	0.02	1.00	0.02
Occupation (rf. professionals)						
Missing	0.91^*^	0.04	0.96	0.04	0.97	0.04
Managers	1.00	0.02	1.02	0.02	1.01	0.02
Technicians	0.91^**^	0.03	0.96	0.03	0.95	0.03
Clerical support workers	1.02	0.03	1.09^**^	0.03	1.09^***^	0.03
Service and sales workers	0.87^***^	0.03	0.94^*^	0.03	0.96	0.03
Tradespeople	0.78^***^	0.04	0.84^**^	0.05	0.89^*^	0.05
Education of father (rf. sec. II)						
Missing			0.94	0.07	0.91	0.07
Secondary education I or less			1.11^**^	0.04	1.09^**^	0.03
Postsecondary			1.00	0.02	1.01	0.02
Tertiary			0.98	0.02	1.00	0.02
Education of mother (rf. sec. II)						
Missing			1.04	0.08	1.06	0.09
Secondary education I or less			1.03	0.03	1.03	0.03
Postsecondary			1.01	0.03	1.01	0.03
Tertiary			1.02	0.02	1.01	0.02
Father: No. of foreign languages			1.05^***^	0.01	1.05^***^	0.01
Mother: No. of foreign languages			1.01	0.01	1.00	0.01
Education (rf. secondary ed.)						
Postsecondary			1.04	0.03	1.03	0.03
Tertiary I			1.09^**^	0.03	1.08^***^	0.03
Tertiary II			1.13^***^	0.04	1.09^**^	0.03
Highbrow cultural orientation			1.03^***^	0.01	1.02^***^	0.01
Family of language (rf. main language)						
Indo-Germanic					0.71^***^	0.03
Other					0.58^***^	0.04
No. of trips to Eu. co. with main lang.					1.01	0.01
No. of stays in countries with main lang.					1.04^**^	0.01
Language of partner (rf. same as ego)						
One of the Swiss main languages					1.15^***^	0.03
Other language family					0.99	0.02
Percentage of Swiss friends					1.00	0.00
Social network within the EU					1.05^*^	0.02
Social network outside of the EU					1.01	0.02
English native speaker (rf. no)					0.95	0.07
Multilingual (rf. no)					1.04^*^	0.02
Percent of persons with same language					0.998^***^	0.00
Highest qualification in Switzerland					1.16^***^	0.03
Intercept	2.05^***^	0.05	1.65^***^	0.06	1.52^***^	0.10
Chi^2^	335.99		521.75		923.53	
Pseudo-R^2^ (Nagelkerke)	0.06		0.07		0.10	
AIC	5408.65		5410.20		5382.21	
BIC	5481.00		5560.48		5604.84	
N	1931.00		1931.00		1931.00	

*Source: Data from the Swiss EUMARR survey*;

* *p < 0.05*;

** *p < 0.01*;

*** *p < 0.001*.

As was also evident descriptively, migrants and non-migrants in our sample did not differ significantly in their endowment with transnational linguistic capital (cf. Table 3). The traditional assimilation view of migrants usually marks them as “deficient” in certain respects. Our study shows that migrants may have as much transnational linguistic capital as non-migrants – although the market value of the languages spoken may vary. The positive effect of Swiss with a migrant background indicating that on average they speak more foreign languages than the autochthonous population in Zurich became insignificant as soon as the language context was controlled (cf. model 3a). This is mainly due to the strong positive effect of multilingual upbringing.

Since the results for both forms of linguistic capital are very similar, we first discuss the similarities, followed by expected differences. Turning to the intergenerational reproduction of cultural capital, we find that it is primarily the number of languages a father spoke when the respondent was a child that has a significant positive effect, which partly supports H_2_ (cf. models 2a and 2b). Parental education has no direct effect on linguistic capital (H_1_) when controlling for the other variables on the intergenerational reproduction of cultural capital. Only adding parental education to model 1a and 1b would show that especially higher education of the father has a significant positive effect (results are not shown, but available from the authors). In contrast, respondents' education has a strong and significant association with linguistic capital (H_4_), which is assumed to be a result of both increased exposure and increased efficiency coming with higher education. Moreover, our study demonstrates that a highbrow cultural orientation has a consistent positive association with linguistic capital (H_3_). As assumed by Bourdieu, different forms of cultural capital cohere, and highbrow cultural capital still seems to be an important part of contemporary cultural capital. A comparison of model 1 and 2 indicates, that the variables derived from this theoretical discussion increase the explanatory power of the models. However, the increase is clearly stronger in the case of transnational linguistic capital.

Due to the differences in the results, the variables added in model 3 will be discussed separately for transnational and Swiss-specific linguistic capital. For the former, the hypothesis relating to transnational experiences and relations (H_5_) receives strong support for transnational linguistic capital (cf. model 3a). All five indicators have a positive impact on the transnational linguistic repertoire. A particularly important factor is having a partner with a different mother tongue, since having such a partner also shapes the composition of a person's social network. Moreover, we find that the indicators of language and migration context largely correspond to expectations: growing up in a bilingual or multilingual family is significantly associated with greater linguistic capital, as is having acquired their highest educational qualification in Switzerland (H_8_). However, being an English native speaker is associated with significantly less linguistic capital. This could be explained by the fact that speaking English as a lingua franca is sufficient to get by in an international city like Zurich. The lack of incentives to expand the language repertoire would thus outweigh the effect of exposure. The share of people who speak the same mother tongue as the respondent has a significant negative, but negligibly small effect on linguistic capital, which may be due to the composition of our sample (many of the respondents' languages are spoken as mother tongues, second or foreign languages by the inhabitants of Zurich). Both results are consistent with hypothesis 7.

With respect to the determinants of acquiring Swiss-specific linguistic capital, we will highlight notable results (cf. Table 4). As hypothesized, foreigners are less likely to speak one or more of the national languages than Swiss (H_6_). Obviously, this applies above all to foreigners whose mother tongue differs from one of the Swiss national languages. In line with this result, obtaining the highest qualifications in Switzerland increases this linguistic capital (H_8_). Exposure to the national languages, as shown by stays abroad in corresponding countries or the partner's mother tongue or social relations within the EU, has the hypothesized positive effect. Travels to and stays in (European) countries where one of the main languages of Switzerland is spoken might differ for the acquisition of Swiss-specific linguistic capital for Swiss and foreigners whose mother tongue is identical to one of those languages. In the case of the foreigners, these trips and stays might concern their home countries, which would not encourage learning of another main Swiss language. In order to take this into account, we also ran a model with interaction effects. While the effects were somewhat stronger for the autochthonous Swiss, the interaction effects were not significant (results available on request). This can be attributed to the particular situation of Switzerland (i.e., size and location in Europe) and the immediate proximity to neighboring countries where the main Swiss languages are spoken, which is used for visits and stays abroad by Swiss and migrants alike. In general, the variables added in model 3 clearly increase the explanatory power of the models in comparison to the models including only the control variables and the variables based on Bourdieu's theory of reproduction. The increase is again stronger for the case of transnational linguistic capital.

We also ran the Poisson regression models separately for persons with and without migration background (see Tables A5–A8 in the Appendix in Supplementary Material). As expected, the results are overall similar. Three differences between the results for Swiss citizens and for foreigners should be highlighted: (1) There are few significant covariations between occupation and linguistic proficiency anyway, however, they differ between Swiss and foreigners. (2) The variables “highest qualification in Switzerland” and “percent of persons with the same language” show significant results in model 3 for foreigners, but not for Swiss. This is due to the fact, that for both variables the variation for Swiss is, for obvious reasons, rather small. (3) Regarding transnational activities and experiences we find somewhat different results for Swiss and foreigners. For Swiss, the number of trips to European countries covaries significantly with the language repertoire - this is not true for foreigners. For social networks in the EU it is the other way around: they are significantly correlated with linguistic proficiency for foreigners but not for Swiss. However, most effects do not differ between the two groups. Thus, the determinants of language repertoire among migrants and autochthonous persons do not differ to a strong degree, and our strategy to combine both groups in one model is justified.

It should be noted that the explanatory power of the models is rather low, which partly results from the small number of cases in relation to the number of covariates in the Poisson regressions. The main reason is, however, that the pseudo-R^2^ measure underestimates the explanatory power of the model compared to the R^2^ measure in ordinary regression models. Andress et al. (1997, p. 288) consider pseudo-R^2^ values between 0.05 and 0.20 to indicate an explanatory power of medium level. However, it could also indicate that important variables were not accounted for.

## Summary and Discussion

Our starting point was that foreign language proficiency is an unequally distributed form of linguistic capital that is becoming more and more crucial in contemporary societies, both for migrants and transnationally active persons. Accordingly, we looked at the determinants of linguistic capital in a broad perspective, combining both the literature on foreign languages as new capital in the era of globalization and Europeanisation (Fligstein, 2008; Gerhards, 2010) and research on migrant integration (Van Tubergen and Kalmijn, 2005; Van Tubergen and Wierenga, 2011). We drew mainly on two theoretical discussions because they point to variables on which not much research exists: The first was the sociological theory of reproduction by Pierre Bourdieu to highlight the structural inequalities underlying the unequal distribution of foreign language proficiency. This theory was up to now rarely taken into account in quantitative research on language learning (Gerhards, 2010; Rössel and Schroedter, 2014). Additionally, since proficiency in the languages of their destination countries is especially important for migrants' structural integration into the labor market and occupational structure, we further added theoretical concepts and hypotheses derived from migration research, focusing especially on the ongoing discussion on the role of transnational ties and experiences for integration into the host country. With respect to this approach our data included a rich set of different measurements of such experiences and relations, thus going beyond existing empirical research. Due to its role as standard explanatory model, we took the economic approach to language, which conceptualizes foreign language proficiency as a kind of human capital, as a source of control variables into account.

We found strong support for the hypotheses derived from the sociological theory of reproduction. Education, both parents' and respondents' own education proved to be important determinants of linguistic capital. However, our empirical results showed that the effect of parental education was mediated entirely by parental linguistic proficiency. This is similar to several studies on the reproduction of cultural capital (Sullivan, 2001; Rössel and Beckert-Zieglschmid, 2002). Furthermore, a highbrow cultural orientation was a consistent indicator of linguistic capital, thus clearly demonstrating the coupling of different forms of cultural capital and the role adherence to the dominant societal culture plays. Overall, our results show that acquiring linguistic capital is deeply embedded into the intergenerational reproduction of inequality in society. Thus, this perspective is clearly important in embedding the investment-oriented economic perspective into the structures of social inequality that underlie acquiring linguistic capital. This does not necessarily contradict the economics of language approach, but highlights the fact that linguistic capital is an unequally distributed resource not only because of different investments in language learning, but because of a strong inequality in the opportunities for language learning. This is a point that social scientific research on language proficiency should definitively take into account.

Finally, we derived a set of hypotheses from migration and integration research. Here, it turned out that education in Switzerland had clearly positive effects on linguistic capital, whereas the results for a migration background proved to be mixed. Although foreigners (i.e., mostly migrants of the first generation) exhibited a lower proficiency in the Swiss national languages, there was no difference with regard to foreign language repertoire in general. This is an important finding because it shows that while migrants may have a deficit in the national languages of their destination country, they do not necessarily have a deficit in foreign language skills in general, suggesting that a deficit-oriented approach can overlook part of migrants' life situations. Furthermore, the opportunity to speak one's mother tongue was significantly related to speaking fewer national languages.

In terms of transnational experiences and relationships, we found quite clear-cut results. They undoubtedly promote foreign language proficiency and increase proficiency in national languages if these experiences or relations are related to countries where the national languages are spoken. Thus, our empirical findings demonstrate that transnational experiences are relevant for linguistic proficiency both for persons with and without migration background and should be included in a social scientific explanation. The empirical results differ somewhat for persons with and without migration background, thus it is very important to include a differentiated set of measuremets of such experiences and relations. However, most transnational activities, like traveling, spending times in foreign countries and meeting friends abroad, are based on the availability of socioenomic resources, thus again indicating that language learning is embedded into the existing social structures of inequality (Itzigsohn and Saucedo, 2002; Guarnizo et al., 2003; Portes et al., 2003; Fligstein, 2008; Gerhards, 2010).

Our empirical results indicate that the variables suggested by the sociology of reproduction and the discussion on migration and integration strongly contribute to the explanatory power of the statistical models of language proficiency. However, these variables should be integrated into a unified approach to language proficiency, e.g., the economic approach to language. The variables suggested by the sociology of reproduction (education of parents, linguistic capital of parents, own education, highbrow culture) focus mainly on the opportunities and the efficiency of language learning, seen through the lenses of the economic approach. In contrast to the economic approach with its focus on language learning as an investment, the sociology of reproduction emphasizes that especially the opportunities to learn languages are unequally distributed and thus the constraints for individual choices. Also, transnational relations and opportunities mainly increase the opportunity to learn foreign languages, but they are also based on unequally distributed resources. Hence, a next step in the development of theories of language acquisition should be to integrate these variables into a coherent model that takes the inequality in opportunities and constraints into account. A very promising framework has been developed by Esser (2006a). Similar to the economic approach, he focuses on the motivation, opportunity, costs, and efficiency as determinants of language learning. However, in contrast to the economic approach, he goes beyond a mere focus on economic variables and takes social and cultural variables comprehensively into account.

Finally, we must discuss some limitations of our study. On the one hand, as outlined above, the measurement of linguistic proficiency could be more precise. Future research should focus on testing the robustness of our empirical results concerning the determinants of linguistic capital by including different measurements of language proficiency. Another weakness concerns a possible selection bias, as the questionnaire was only available in German or English and participation was therefore limited to people who were able to understand and read those languages. In addition, our sample included a very specific selection of persons with and without migration background in a very specific country, i.e., Switzerland. Thus, to generalize the empirical results regarding the sociology of reproduction and the transnationalisation literature, further cases need to be studied. Furthermore, our research design completely relies on cross-sectional data. Thus, we cannot interpret most of our results in a causal manner, but have to interpret them as empirical covariations. Yet, to the extent that the empirical correlations support our hypotheses, they suggest that our theoretical approaches lead to correct hypotheses and are therefore not falsified. A stronger interpretation is possible for the role of parental education and parental lingustic capital. Since there is a plausible time ordering involved, these covariations can be interpreted in a causal manner with a higher degree of certainty and thus support the role unequal opportunities play in learning languages. However, in order to present stronger arguments for a causal interpretation, future studies should rely on longitudinal empirical designs.

## Data Availability Statement

The raw data supporting the conclusions of this article will be made available by the authors, without undue reservation.

## Ethics Statement

Ethical review and approval was not required for the study on human participants in accordance with the local legislation and institutional requirements. The patients/participants provided their written informed consent to participate in this study.

## Author Contributions

All authors listed have made a substantial, direct and intellectual contribution to the work, and approved it for publication.

## Conflict of Interest

The authors declare that the research was conducted in the absence of any commercial or financial relationships that could be construed as a potential conflict of interest.

## Footnotes

^1^This information is based on the Swiss Job Market Monitor: https://www.stellenmarktmonitor.uzh.ch/en.html. We thank Ann-Sophie Gnehm for analysing the data.

^2^The arguments of the economic approach apply not only to migrants, but also to the foreign language proficiency of autochthonous populations, e.g., Swiss-German persons learning a further national language or a foreign language.

^3^In our discussion, we mainly focus on the incorporated cultural capital. According to Bourdieu, the cultural capital can also take two other forms: that of institutionalized cultural capital in the form of educational titles and that of objectified cultural capital in the form of objects (e.g., books or musical instruments).

^4^Visits to “home countries” are crucial for maintaining the language of origin. For instance, frequent visits to their parents' country of origin have been shown to be a major factor in second-generation migrants' proficiency in their parents' mother tongue (Soehl, 2018, p. 1529).

^5^The sample was restricted to couples within a specific age range (men between 30 and 45 years of age, with partners aged between 18 and 57) and with certain citizenships; for more details, see Schroedter and Rössel (2013).

^6^In detail the question read as follows: “Please specify all the languages you currently speak apart from your mother tongue.” It was complemented with the clarification: “By speaking a language we mean that you can have a long conversation in another language with native speakers of that language.”

^7^We ran Poisson regressions because our outcome variables were count data, and Poisson regressions are the best choice for modeling this kind of data. In order to control for mild violations of underlying assumptions, we used robust standard errors as recommended by Cameron and Trivedi (2010, p. 574).

^8^Table A3 provides an overview of the distribution of the different variables (cf. Appendix).

## References

[B1] Alba R. Logan J. Lutz A. Stults B. (2002). Only english by the third generation? Demography 39, 467–484. 10.1353/dem.2002.0023 12205753

[B2] Amelina A. (2010). Transnationale Migration jenseits von Assimilation und Akkulturation. Berliner Z. Soziol. 20, 257–279. 10.1007/s11609-010-0123-y

[B3] Andres M. Korn K. Barjak F. Glas A. Leukens A. Niederer R. (2005). Fremdsprachen in Schweizer Betrieben. Olten: Fachhochschule Nordwestschweiz.

[B4] Andress H.-J. Hagenaars J. A. Kühnel S. (1997). Analyse von Tabellen und kategorialen Daten: log-lineare Modelle, latente Klassenanalyse, logistische Regression und GSK-Ansatz. Berlin: Springer. 10.1007/978-3-662-05693-6

[B5] BfS (Bundesamt für Statistik) (2018). Sprachen bei der Arbeit. Erhebung zur Sprache, Religion und Kultur 2014. Available online at: https://www.bfs.admin.ch/bfs/de/home/statistiken/bevoelkerung/sprachen-religionen/sprachen.assetdetail.4902599.html (accessed Febuary 2, 2021).

[B6] Birdsong D. (2006). Age and second language acquisition and processing: a selective overview. Lang. Learn. 56, 9–49. 10.1111/j.1467-9922.2006.00353.x

[B7] Bourdieu P. (1970). Zur Soziologie der Symbolischen Formen. Frankfurt/Main: Suhrkamp.

[B8] Bourdieu P. (1982). Die feinen Unterschiede. Kritik der gesellschaftlichen Urteilskraft. Frankfurt/Main: Suhrkamp.

[B9] Bourdieu P. (1990). Was heißt Sprechen? Die Ökonomie des sprachlichen Tausches. Wien: Braumüller.

[B10] Bourdieu P. Passeron J. C. (1977). Reproduction in Education, Society and Culture. London: Sage.

[B11] Braun M. (2010). Foreign language proficiency of intra-European migrants: a multilevel analysis. Eur. Sociol. Rev. 26, 603–617. 10.1093/esr/jcp052

[B12] Cameron A. C. Trivedi P. K. (2010). Microeconometrics Using Stata. Revised Edn. College Station, TX: Stata Press.

[B13] Charette M. Meng R. (1994). Explaining language proficiency: objective versus self-assessed measures of literacy. Econ. Lett. 44, 313–321. 10.1016/0165-1765(93)00365-U

[B14] Chiswick B. (2008). The economics of language: an introduction and overview, in IZA Discussion Paper 3568 (Bonn: IZA).

[B15] Chiswick B. Miller P. W. (1994). Language choice among immigrants in a multi-lingual destination. J. Popul. Econ 7, 119–131. 10.1007/BF00173615 12345481

[B16] Chiswick B. Miller P. W. (2001). A model of destination-language acquisition: application to male immigrants in Canada. Demography 38, 391–409. 10.1353/dem.2001.0025 11523267

[B17] Chiswick B. Miller P. W. (2007). Matching language proficiency to occupation: the effect on immigrants' earnings, in IZA Discussion Paper 2587 (Bonn: IZA).

[B18] Chiswick B. Miller P. W. (2014). International migration and the economics of language, in IZA Discussion Paper 7880 (Bonn: IZA).

[B19] Dahinden J. (2009). Are we all transnationals now? Network transnationalism and transnational subjectivity: the differing impacts of globalization on the inhabitants of a small Swiss city. Ethn. Racial Stud. 32, 1365–1386. 10.1080/01419870802506534

[B20] Ebner C. Helbling M. (2016). Social distance and wage inequalities for immigrants in Switzerland. Work Employ. Soc. 30, 436–454. 10.1177/0950017015594096

[B21] EDA (2021). Personenfreizügigkeit. Available online at: www.eda.admin.ch (accessed Febuary 2, 2021).

[B22] EDK (2021). Fremdsprachen: Sprache, Beginn. Available online at: www.edk.ch (accessed Febuary 2, 2021).

[B23] Esser H. (2006a). Sprache und Integration. Die sozialen Bedingungen und Folgen des Spracherwerbs von Migranten. Frankfurt: Campus.

[B24] Esser H. (2006b). Ethnische Ressourcen: das Beispiel der Bilingualität. Berliner J. Soziol. 16, 89–104. 10.1007/s11609-006-0220-0

[B25] Esser H. (2008). Spracherwerb und Einreisealter: Die schwierigen Bedingungen der Bilingualität, in Migration und Integration, Sonderheft 48 der Kölner Zeitschrift für Soziologie und Sozialpsychologie, ed Kalter F. (Wiesbaden: VS), 202–229.

[B26] Fligstein N. (2008). Euroclash. Oxford: Oxford University Press. 10.1093/acprof:oso/9780199580859.001.0001

[B27] Gerhards J. (2010). Mehrsprachigkeit im vereinten Europa. Transnationales sprachliches Kapital als Ressource in einer globalisierten Welt. Wiesbaden: VS. 10.1007/978-3-531-92036-8

[B28] Grin F. (1999). Compétences et Récompenses. La valeur des Langues en Suisse. Freiburg: Éditions Universitaires.

[B29] Guarnizo L. Portes A. Haller W. (2003). Assimilation and transnationalism. Determinants of transnational political action among contemporary migrants. Am. J. Sociol. 108, 1211–1248. 10.1086/375195

[B30] Hufeisen B. Riemer C. (2010). Spracherwerb und Sprachenlernen, *in Deutsch als Fremd- und Zweitsprache. Ein internationales Handbuch*, eds Krumm H. P. Fandrych C. Hufeisen B. Riemer C. (Berlin: de Gruyter), 738–753.

[B31] Isphording I. E. Otten S. Sinning M. (2014). The effect of language deficiency on immigrant labor market outcomes in Germany, in Paper presented at the 11th SOEP User Conference (Berlin: DIW).

[B32] Itzigsohn J. Saucedo S. G. (2002). Immigrant incorporation and sociocultural transnationalism. Int. Migr. Rev. 36, 766–798. 10.1111/j.1747-7379.2002.tb00104.x

[B33] Jacob K. Kalter F. (2011). Die intergenerationale Transmission von hochkulturellen Lebensstilen unter Migrationsbedingungen, in Lebensstilforschung. Sonderband 51 der Kölner Zeitschrift für Soziologie und Sozialpsychologie, eds Rössel J. Otte G. (Wiesbaden: VS), 223–246.

[B34] Jaeger M. M. Breen R. (2016). A dynamic model of cultural reproduction. Am. J. Sociol. 121, 1079–1115. 10.1086/684012 27017707

[B35] Jude N. (2008). Zur Struktur von Sprachkompetenz (Dissertation). Frankfurt/Main. Available online at: http://publikationen.ub.uni-frankfurt.de/frontdoor/index/index/docId/6694 (accessed Febuary 2, 2021).

[B36] Mau S. (2010). Social Transnationalism. Lifeworlds beyond the Nation State. London; New York, NY: Routledge. 10.4324/9780203879061

[B37] Portes A. Guarnizo L. Haller W. J. (2003). Transnational entrepreneurs: an alternative form of immigrant economic adaptation. Am. Sociol. Rev. 67, 278–298. 10.2307/3088896

[B38] Rössel J. Beckert-Zieglschmid C. (2002). Die Reproduktion kulturellen Kapitals. Z. Soziol. 31, 497–513. 10.1515/zfsoz-2002-0603

[B39] Rössel J. Schroedter J. H. (2014). Der Erwerb linguistischen Kapitals. Transnationales und Schweiz-spezifisches linguistisches Kapital im Vergleich, in Globalisierung, Bildung und grenzüberschreitende Mobilität, eds Gerhards J. Hans S. Carlson S. (Wiesbaden: Springer VS), 153–183. 10.1007/978-3-658-02439-0_8

[B40] Schans D. (2009). Transnational family ties of immigrants in the Netherlands. Ethn. Racial Stud. 32, 1164–1182. 10.1080/01419870902763852

[B41] Schroedter J. H. Rössel J. (2013). Codebook of the EUMARR Survey: Switzerland. Available online at: https://www.suz.uzh.ch/de/institut/mitarbeitende/schroedter/forschung/eumarr.html (accessed June 02, 2020).

[B42] Snel E. Engbersen G. Leerkes A. (2006). Transnational involvement and integration. Global Netw. 6, 285–308. 10.1111/j.1471-0374.2006.00145.x

[B43] Soehl T. (2018). But do they speak it? The intergenerational transmission of home-country language in migrant families in France. J. Ethn. Migr. Stud. 42, 1513–1535. 10.1080/1369183X.2015.1126171

[B44] Soehl T. Waldinger R. (2010). Making the connection: Latino immigrants and their cross-border ties. Ethn. Racial Stud. 33, 1489–1510. 10.1080/01419871003624050

[B45] Stevens G. (1985). Nativity, intermarriage, and mother-tongue shift. Am. Sociol. Rev. 50, 74–83. 10.2307/2095341

[B46] Sullivan A. (2001). Cultural capital and educational attainment. Sociology 35, 893–912. 10.1177/0038038501035004006

[B47] Teney C. Deutschmann E. (2018). Transnational social practices: a quantitative perspective, in Emerging Trends in the Social and Behavioral Sciences, eds Scott R. Buchmann M. (Hoboken: John Wiley and Son), 1–15. 10.1002/9781118900772.etrds0456

[B48] Van Tubergen F. Kalmijn M. (2005). Destination-language proficiency in cross-national perspective: a study of immigrant groups in nine Western countries. Am. J. Sociol. 110, 1412–1457. 10.1086/428931

[B49] Van Tubergen F. Kalmijn M. (2009). Language proficiency and usage among immigrants in the Netherlands: incentives or opportunities? Eur. Sociol. Rev. 25, 169–182. 10.1093/esr/jcn043

[B50] Van Tubergen F. Wierenga M. (2011). The language acquisition of male immigrants in a multilingual destination. Turks and Moroccans in Belgium. J. Ethn. Migr. Stud. 37, 1039–1057. 10.1080/1369183X.2011.572476

[B51] Werlen I. (2008). Schlussbericht. Sprachkompetenzen der erwachsenen Bevölkerung in der Schweiz. Institute for Linguistics; University of Bern. Available online at: http://www.snf.ch/SiteCollectionDocuments/nfp/nfp56/nfp56_schlussbericht_werlen.pdf (accessed Febuary 2, 2021).

