# Supplementary material for: The Unequal Distribution of Linguistic Capital in a Transnational Economic Order
Source: Front Sociol. 2021 Apr 1;6:568962. doi: 10.3389/fsoc.2021.568962 (PMC8022684; doi:10.3389/fsoc.2021.568962)
Supplement: Supplementary file 1 [file Data_Sheet_1.pdf]

Appendix

Table A1. Most-spoken languages within transnational linguistic capital, by group.

	Fluently spoken additional (foreign) languages			
	Swiss	Swiss with migration background	Foreigners	Total
English	96 %	89 %	85 %	90 %
French	80 %	74 %	45 %	63 %
Italian	30 %	35 %	18 %	27 %
Spanish	22 %	22 %	19 %	21 %
Portuguese	4 %	3 %	3 %	3 %
German*	3 %	21 %	42 %	22 %
Russian	2 %	2 %	4 %	3 %
Total	*697*	*532*	*702*	*1,931*

Source: Data from the Swiss EUMARR survey; * Swiss and High German

Table A2. Most-spoken languages within Swiss-specific linguistic capital, by group.

	Fluently spoken national languages				
	Swiss	Swiss with migration background	Foreigners with a main language as mother tongue	Foreigners with other mother tongue	Total
German*	100 %	99 %	97 %	87 %	97 %
French	80 %	75 %	60 %	30 %	67 %
Italian	31 %	45 %	34 %	17 %	34 %
Total	*697*	*532*	*419*	*283*	*1,931*

Source: Data from the Swiss EUMARR survey; * Swiss and High German (incl. Rhaeto-Romanic)

Table A3. Distribution of the variables used in the models.

Variable	Mean	Std. Dev.	Minimum	Maximum
Number of foreign languages	***2.50***	***1.00***	***0***	***4***
Number of main languages*	***1.98***	***0.76***	***0***	***3***
Age	37.07	5	22	56
Sex (female=1)	0.56	0.50	0	1
Migration background				
Swiss	0.36	0.48	0	1
Swiss with migration background	0.28	0.45	0	1
Foreigner (Swiss main languages as mother tongue)	0.22	0.41	0	1
Foreigner (other mother tongues)	0.15	0.35	0	1
Currently employed	0.76	0.43	0	1
Occupation				
Missing	0.07	0.25	0	1
Managers	0.18	0.39	0	1
Professionals	0.38	0.49	0	1
Technicians	0.10	0.31	0	1
Clerical support workers	0.12	0.33	0	1
Service and sales workers	0.10	0.30	0	1
Tradespeople	0.04	0.20	0	1
Education of father				
Missing	0.02	0.14	0	1
Secondary education I or less	0.13	0.34	0	1
Secondary education II	0.35	0.48	0	1
Postsecondary	0.16	0.36	0	1
Tertiary	0.34	0.47	0	1
Education of mother				
Missing	0.02	0.13	0	1
Secondary education I or less	0.18	0.39	0	1
Secondary education II	0.49	0.50	0	1
Postsecondary	0.12	0.32	0	1
Tertiary	0.19	0.39	0	1
Father: no. of foreign languages	1.47	1.18	0	5
Mother: no. of foreign languages	1.47	1.15	0	5
Education				
Secondary education or less	0.23	0.41	0	1
Postsecondary	0.17	0.37	0	1
Tertiary I	0.48	0.50	0	1
Tertiary II	0.12	0.33	0	1
Highbrow cultural orientation	1.47	1.40	0	5
English native speaker (rf. no)	0.04	0.20	0	1
Raised bi-/multilingual (rf. no)	0.23	0.42	0	1
Highest qualification in Switzerland (rf. no)	0.66	0.47	0	1

Variable	Mean	Std. Dev.	Minimum	Maximum
Percent of persons with same language	47.38	29.64	0.2	67.3
No. of trips to European countries	12.27	4.79	0	32
No. of stays in different countries	1.38	1.22	0	7
Partner with different mother tongue	0.49	0.50	0	1
Social network within the EU	0.73	0.44	0	1
Social network outside of the EU	0.27	0.45	0	1
Family of language				
Main language	0.18	0.38	0	1
Indo-Germanic				
Other	0.03	0.16	0	1
No. of trips to Europ. countries with main lang.	4.35	1.04	0	5
No. of stays in countries with main language	0.50	0.70	0	4
Language of partner				
Same as individual				
One of the Swiss main languages	0.23	0.42	0	1
Other language family	0.26	0.44	0	1
Percentage of Swiss friends	65.64	32.49	0	100

Source: Data from the Swiss EUMARR survey; n=1,931; * Rhaeto-Romanic included in German

Table A4. Means of the variables used in the models over groups.

Variable	Swiss	Swiss with migration back-ground	Foreigners	Mean (total)
Number of foreign languages	***2.54***	***2.75***	***2.37***	***2.50***
Number of main languages*	***2.11***	***2.18***	***1.69***	***1.98***
Age	37.3	37.8	36.3	37.07
Sex (female=1)	0.52	0.61	0.57	0.56
Migration background				0.28
Foreigner (Swiss main languages as mother tongue)	-	-	0.60	0.22
Foreigner (other mother tongues)	-	-	0.40	0.15
Currently employed	0.77	0.76	0.75	0.76
Occupation				
Missing	0.06	0.07	0.07	0.07
Managers	0.19	0.20	0.17	0.18
Professionals	0.39	0.29	44.9	0.38
Technicians	0.12	0.10	0.09	0.10
Clerical support workers	0.13	0.16	0.08	0.12
Service and sales workers	0.08	0.13	0.10	0.10
Tradespeople	0.03	0.05	0.05	0.04
Education of father				
Missing	0.02	0.02	0.02	0.02
Secondary education I or less	0.05	0.19	0.16	0.13
Secondary education II	0.44	0.33	0.27	0.35
Postsecondary	0.20	0.14	0.13	0.16
Tertiary	0.29	0.31	0.42	0.34
Education of mother				
Missing	0.02	0.01	0.02	0.02
Secondary education I or less	0.11	0.26	0.20	0.18
Secondary education II	0.66	0.44	0.37	0.49
Postsecondary	0.12	0.12	0.11	0.12
Tertiary	0.10	0.16	0.31	0.19
Father: no. of foreign languages	1.69	1.72	1.07	1.47
Mother: no. of foreign languages	1.76	1.67	1.04	1.47
Education				
Secondary education or less	0.23	0.31	0.16	0.23
Postsecondary	0.20	0.19	0.11	0.17
Tertiary I	0.47	0.39	0.56	0.48
Tertiary II	0.10	0.09	0.18	0.12
Highbrow cultural orientation	1.45	1.39	1.56	1.47
English native speaker (rf. no)	-	0.02	0.09	0.04
Raised bi-/multilingual (rf. no)	0.09	0.43	0.21	0.23

**Variable**	**Swiss**	**Swiss with migration back-ground**	**Foreigners**	**Mean (total)**
Highest qualification in Switzerland (rf. no)	0.93	0.82	0.26	0.66
Percent of persons with same language	64.70	44.57	32.31	47.38
No. of trips to European countries	12.72	11.75	12.21	12.27
No. of stays in different countries	1.13	1.19	1.74	1.38
Partner with different mother tongue	0.31	0.50	0.66	0.49
Social network within the EU	0.55	0.74	0.91	0.73
Social network outside of the EU	0.22	0.30	0.30	0.27
Family of language				
Main language	-	0.79	0.60	0.80
Indo-Germanic	-	0.22	0.36	0.18
Other	-	0.29	0.04	0.03
No. of trips to Europ. countries with main lang.	4.55	4.29	4.20	4.35
No. of stays in countries with main language	0.20	0.43	0.86	0.50
Language of partner				
Same as individual	0.69	0.50	0.34	0.51
One of the Swiss main languages	0.04	0.22	0.42	0.23
Other language family	0.27	0.28	0.24	0.26
Percentage of Swiss friends	84.76	70.05	43.29	65.64
Total (n)	***697***	***532***	***702***	***1,931***

Source: Data from the Swiss EUMARR survey

Table A5. Determinants of the acquisition of transnational linguistic capital – only for Swiss citizens.

	Model 1a		Model 2a		Model 3a	
	IRR	R.SE	IRR	R.SE	IRR	R.SE
Age (cent. 37 years)	1.00	0.00	1.00	0.00	1.00	0.00
Sex (rf. male)	1.16***	0.04	1.16***	0.04	1.17***	0.04
Employed	1.01	0.03	0.98	0.03	0.96	0.03
Occupation (rf. professionals)						
Missing	0.80**	0.06	0.91	0.06	0.90	0.05
Managers	0.92*	0.04	0.98	0.04	0.94	0.04
Technicians	0.79***	0.04	0.87*	0.05	0.87**	0.04
Clerical support workers	0.92	0.04	1.06	0.05	1.02	0.05
Service and sales workers	0.76***	0.05	0.90	0.06	0.90	0.06
Tradespeople	0.62***	0.09	0.76*	0.10	0.77*	0.10
Education of father (rf. sec. II)						
Missing			0.86	(0.15)	0.83	(0.17)
Secondary education I or less			0.99	(0.10)	0.99	(0.09)
Postsecondary			1.03	(0.04)	1.03	(0.04)
Tertiary			0.99	(0.04)	0.98	(0.04)
Education of mother (rf. sec. II)						
Missing			1.07	(0.17)	1.07	(0.20)
Secondary education I or less			0.98	(0.06)	0.98	(0.05)
Postsecondary			1.04	(0.04)	1.04	(0.04)
Tertiary			1.03	(0.05)	0.99	(0.05)
Father: no. of foreign languages			1.08***	(0.02)	1.09***	(0.02)
Mother: no. of foreign languages			1.00	(0.02)	0.99	(0.01)
Education (rf. secondary ed.)						
Postsecondary			1.04	(0.06)	1.04	(0.05)
Tertiary I			1.19***	(0.06)	1.15**	(0.05)
Tertiary II			1.29***	(0.08)	1.24***	(0.07)
Highbrow cultural orientation			1.02*	(0.01)	1.01	(0.01)
Multilingual (rf. no)					1.11*	(0.06)
Highest qualification in Switzerland					1.03	(0.06)
Percent of persons with same language					1.00	(0.00)
No. of trips to European countries					1.01*	(0.00)
No. of stays in different countries					1.07***	(0.01)
Partner with different mother tongue					1.18***	(0.04)
Social network within the EU					1.02	(0.03)
Social network outside of the EU					1.06	(0.03)
Intercept	2.47***	(0.10)	1.75***	(0.12)	1.46***	(0.16)
Chi^2	73.81		174.11		294.41	
Pseudo-R^2 (Nagelkerke)	0.05		0.09		0.13	

	Model 1a		Model 2a		Model 3a	
	IRR	R.SE	IRR	R.SE	IRR	R.SE
AIC	2196.01		2192.80		2179.85	
BIC	2241.48		2301.93		2325.34	
N	697		697		697	

Source: Data from the Swiss EUMARR survey; * p<0.05; ** p<0.01; *** p<0.001

Table A6. Determinants of the acquisition of transnational linguistic capital – without Swiss citizens.

	Model 1a		Model 2a		Model 3a	
	IRR	R.SE	IRR	R.SE	IRR	R.SE
Age (cent. 37 years)	1.00	0.00	1.00	0.00	1.00	0.00
Sex (rf. male)	1.07^{**}	0.02	1.07^{**}	0.02	1.07^{***}	0.02
Migration background (rf. Foreigner)						
Swiss with migration background	1.15^{***}	0.03	1.10^{***}	0.03	1.04	0.03
Currently employed (rf. not employed)						
Employed	1.00	0.03	0.99	0.03	0.97	0.02
Occupation (rf. professionals)						
Missing	1.02	0.05	1.09	0.05	1.05	0.05
Managers	1.06	0.03	1.07^{*}	0.03	1.04	0.03
Technicians	0.98	0.04	1.03	0.04	1.00	0.04
Clerical support workers	1.06	0.04	1.15^{***}	0.04	1.13^{***}	0.04
Service and sales workers	0.88^{**}	0.04	0.96	0.04	0.96	0.04
Tradespeople	0.81^{**}	0.06	0.87	0.07	0.89	0.07
Education of father (rf. sec. II)						
Missing			0.98	0.08	0.95	0.08
Secondary education I or less			1.06	0.04	1.02	0.04
Postsecondary			0.99	0.04	1.01	0.04
Tertiary			0.96	0.03	0.97	0.03
Education of mother (rf. sec. II)						
Missing			1.09	0.11	1.11	0.11
Secondary education I or less			1.07^{*}	0.04	1.04	0.03
Postsecondary			1.01	0.04	0.98	0.03
Tertiary			1.04	0.03	1.00	0.03
Father: no. of foreign languages			1.07^{***}	0.01	1.06^{***}	0.01
Mother: no. of foreign languages			1.03^{*}	0.01	1.02	0.01
Education (rf. secondary ed.)						
Postsecondary			1.07	0.04	1.05	0.04
Tertiary I			1.15^{***}	0.04	1.14^{***}	0.04
Tertiary II			1.17^{***}	0.05	1.12^{**}	0.05
Highbrow cultural orientation			1.02^{*}	0.01	1.01	0.01
English native speaker (rf. no)					0.73^{***}	0.04
Multilingual (rf. no)					1.13^{***}	0.02
Highest qualification in Switzerland					1.16^{***}	0.03
Percent of persons with same language					0.999^{***}	0.00
No. of trips to European countries					1.00	0.00
No. of stays in different countries					1.03^{**}	0.01
Partner with different mother tongue					1.11^{***}	0.03
Social network within the EU					1.07^{*}	0.03

	Model 1a		Model 2a		Model 3a	
	IRR	R.SE	IRR	R.SE	IRR	R.SE
Social network outside of the EU					1.03	0.02
Intercept	2.29***	0.08	1.72***	0.09	1.49***	0.10
Chi^2	86.67		249.37		486.66	
Pseudo-R^2 (Nagelkerke)	0.03		0.06		0.10	
AIC	3899.44		3890.50		3850.80	
BIC	3955.74		4018.45		4024.81	
N	1,234		1,234		1,234	

Source: Data from the Swiss EUMARR survey; * p<0.05; ** p<0.01; *** p<0.001

Table A7. Determinants of the acquisition of Swiss-specific linguistic capital – only for Swiss citizens.

	**Model 1b**		**Model 2b**		**Model 3b**	
	IRR	R.SE	IRR	R.SE	IRR	R.SE
Age (cent. 37 years)	1.00	0.00	1.01^{**}	0.00	1.01^{**}	0.00
Sex (rf. male)	1.14^{***}	0.03	1.14^{***}	0.03	1.14^{***}	0.03
Currently employed (rf. not employed)						
Employed	1.02	0.03	1.00	0.03	0.99	0.03
Occupation (rf. professionals)						
Missing	0.85^{**}	0.05	0.93	0.05	0.93	0.05
Managers	0.93^{*}	0.03	0.97	0.03	0.97	0.03
Technicians	0.83^{***}	0.04	0.89^{**}	0.04	0.90^{*}	0.04
Clerical support workers	0.96	0.04	1.06	0.04	1.06	0.04
Service and sales workers	0.83^{***}	0.04	0.94	0.05	0.95	0.05
Tradespeople	0.75^{***}	0.06	0.87	0.06	0.89	0.07
Education of father (rf. sec. II)						
Missing			0.85	0.19	0.82	0.19
Secondary education I or less			1.04	0.07	1.04	0.07
Postsecondary			1.04	0.03	1.04	0.03
Tertiary			0.99	0.03	1.00	0.03
Education of mother (rf. sec. II)						
Missing			1.07	0.22	1.10	0.24
Secondary education I or less			0.98	0.04	0.99	0.04
Postsecondary			1.00	0.03	1.00	0.03
Tertiary			1.02	0.04	1.00	0.04
Father: No. of foreign languages			1.08^{***}	0.01	1.07^{***}	0.01
Mother: No. of foreign languages			0.98	0.01	0.98	0.01
Education (rf. secondary ed.)						
Postsecondary			1.04	0.04	1.04	0.04
Tertiary I			1.13^{***}	0.04	1.13^{***}	0.04
Tertiary II			1.21^{***}	0.06	1.19^{***}	0.05
Highbrow cultural orientation			1.02^{**}	0.01	1.02^{*}	0.01
Multilingual (rf. no)					1.07	0.05
Highest qualification in Switzerland					1.03	0.05
Percent of persons with same language					0.999	0.00
No. of trips to Eu. co. with main lang.					1.03^{*}	0.02
No. of stays in countries with main lang.					1.05^{*}	0.02
Language of partner (rf. same as ego)						
One of the Swiss main languages					1.12^{*}	0.06
Other language family					1.03	0.03

	Model 1b		Model 2b		Model 3b	
	IRR	R.SE	IRR	R.SE	IRR	R.SE
Percentage of Swiss friends					1.00	0.00
Social network within the EU					1.02	0.02
Social network outside of the EU					1.01	0.03
Intercept	2.08***	0.07	1.63***	0.08	1.45**	0.17
Chi^2	84.66		207.12		256.79	
Pseudo-R^2 (Nagelkerke)	0.02		0.05		0.06	
AIC	1986.64		1997.92		2013.29	
BIC	2032.11		2107.04		2167.88	
N	697		697		697	

Source: Data from the Swiss EUMARR survey; * p<0.05; ** p<0.01; *** p<0.001

Table A8. Determinants of the acquisition of Swiss-specific linguistic capital – without Swiss citizens.

	**Model 1b**		**Model 2b**		**Model 3b**	
	IRR	R.SE	IRR	R.SE	IRR	R.SE
Age (cent. 37 years)	1.01***	0.00	1.01**	0.00	1.01**	0.00
Sex (rf. male)	1.06*	0.02	1.05*	0.02	1.05*	0.02
Migration background (rf. foreigner)						
Swiss with migration background	1.58***	0.05	1.54***	0.05	1.24***	0.06
Foreigner with national language	1.40***	0.05	1.40***	0.05	1.13**	0.05
Currently employed (rf. not employed)						
Employed	1.02	0.03	1.00	0.03	1.01	0.02
Occupation (rf. professionals)						
Missing	0.94	0.05	0.96	0.05	1.00	0.05
Managers	1.05	0.03	1.05	0.03	1.03	0.03
Technicians	0.98	0.04	0.99	0.04	0.97	0.04
Clerical support workers	1.07*	0.04	1.11**	0.04	1.10**	0.04
Service and sales workers	0.90*	0.04	0.93	0.04	0.96	0.04
Tradespeople	0.80**	0.06	0.83**	0.06	0.90	0.06
Education of father (rf. sec. II)						
Missing			0.95	0.08	0.91	0.08
Secondary education I or less			1.10*	0.04	1.07*	0.04
Postsecondary			0.96	0.03	0.98	0.03
Tertiary			0.97	0.03	0.99	0.03
Education of mother (rf. sec. II)						
Missing			1.05	0.10	1.09	0.10
Secondary education I or less			1.05	0.04	1.04	0.03
Postsecondary			1.02	0.04	1.03	0.04
Tertiary			1.03	0.03	1.02	0.03
Father: No. of foreign languages			1.03*	0.01	1.03*	0.01
Mother: No. of foreign languages			1.03*	0.01	1.02	0.01
Education (rf. secondary ed.)						
Postsecondary			1.05	0.04	1.02	0.04
Tertiary I			1.05	0.04	1.06	0.03
Tertiary II			1.08	0.05	1.04	0.04
Highbrow cultural orientation			1.03***	0.01	1.02*	0.01
English native speaker (rf. no)					0.96	0.07
Multilingual (rf. no)					1.04	0.02
Highest qualification in Switzerland					1.19***	0.03
Family of language (rf. main language)						
Indo-Germanic					0.71***	0.03
Other					0.58***	0.04

	Model 1b		Model 2b		Model 3b	
	IRR	R.SE	IRR	R.SE	IRR	R.SE
Percent of persons with same language					0.998***	0.00
No. of trips to Eu. co. with main lang.					1.00	0.01
No. of stays in countries with main lang.					1.04**	0.01
Language of partner (rf. same as ego)						
One of the Swiss main languages					1.14***	0.04
Other language family					0.96	0.03
Percentage of Swiss friends					1.00	0.00
Social network within the EU					1.08*	0.03
Social network outside of the EU					1.00	0.02
Intercept	1.32***	0.06	1.12	0.07	1.28**	0.12
Chi^2	277.88		386.60		769.53	
Pseudo-R^2 (Nagelkerke)	0.07		0.08		0.12	
AIC	3434.82		3448.99		3425.27	
BIC	3496.23		3582.06		3624.87	
N	1234		1234		1234	

Source: Data from the Swiss EUMARR survey; * p<0.05; ** p<0.01; *** p<0.001
